# Characteristics of dyslipidemia in adults with Resistance to Thyroid Hormone β

**DOI:** 10.1016/j.eprac.2025.07.001

**Published:** 2025-07-05

**Authors:** Timothy M E Davis, Wendy A Davis, Carla Moran, Greta Lyons, Ellis Bryden, Krishna Chatterjee

**Affiliations:** 1 University of Western Australia, Medical School, Fremantle Hospital, Fremantle 6160, Western Australia, Australia; 2 Department of Endocrinology and Diabetes, Fiona Stanley and Fremantle Hospitals, Murdoch 6150, WA, Australia; 3 Beacon Hospital, Dublin D18 AK68, Ireland; 4 St. Vincent’s University Hospital, Dublin D04 T6F4, Ireland; 5 School of Medicine, University College, Dublin D04 C1P1, Ireland; 6 Institute of Metabolic Science, University of Cambridge, Cambridge CB2 0QQ, UK; 7 Department of Clinical Biochemistry, Addenbrooke’s Hospital, Cambridge CB2 0QQ, UK

**Keywords:** Resistance to Thyroid Hormone β, dyslipidemia, insulin resistance

## Abstract

**Objective:**

To assess determinants of dyslipidemia (specifically raised serum low-density lipoprotein (LDL) cholesterol and triglycerides, and reduced high-density lipoprotein (HDL) cholesterol) in individuals with Resistance to Thyroid Hormone β (RTHβ).

**Methods:**

Multivariable linear regression analyses of cross-sectional fasting serum lipid profiles from 105 genetically-confirmed individuals with RTHβ (mean age 40.0 years, 40% males) were conducted, including candidate variables with plausible independent associations with the individual components of dyslipidemia such as indices of thyroid function, plasma non-esterified fatty acid (NEFA) concentrations, and insulin resistance (IR) assessed using the Homoeostasis Model Assessment (HOMA) equation.

**Results:**

Age was positively related to both serum total and LDL-cholesterol concentrations (*P*<0.001), while there were similar directional associations between body mass index and total:HDL-cholesterol ratio, and HOMA-IR and serum triglycerides (*P*≤0.007). Plasma NEFA concentrations were not associated with serum triglycerides. The only thyroid function measure revealed by the individual models was an inverse association between reverse T3 and serum triglycerides (*P*=0.009).

**Conclusions:**

The dyslipidemia associated with RTHβ shares recognized contributory factors found in studies of the general population but appears independent of thyroid status. The reason for the significant inverse association between circulating reverse T3 and serum triglyceride concentrations is unclear and merits further investigation.

## Introduction

Resistance to Thyroid Hormone β (RTHβ) is an uncommon disorder characterized by elevated circulating thyroid hormones (TH) with non-suppressed TSH levels due typically to heterozygous mutations in thyroid hormone receptor β [1]. RTHβ can be associated with dyslipidemia, specifically raised serum low-density lipoprotein (LDL) cholesterol and triglycerides, and reduced high-density lipoprotein (HDL) cholesterol, relative to concentrations in matched healthy controls [2, 3]. This has been attributed to hepatic resistance to thyroid hormone action, with reduced cell surface expression of the LDL receptor (a recognised thyroid hormone-regulated target gene) mediating lower clearance of circulating LDL-cholesterol, while increased circulating non-esterified fatty acid (NEFA) concentrations are postulated to contribute to increased triglyceride synthesis [2]. In addition, RTHβ is associated with increased systemic insulin resistance (IR) which has a well-recognized relationship with high serum triglyceride and low HDL-cholesterol concentrations [4].

In order to explore relationships between dyslipidemia, NEFA and IR in RTHβ, and thus their implications for clinical management, we analyzed serum lipid and related data from all adult RTHβ patients with diverse thyroid hormone receptor β mutations who had been referred to a UK specialist centre for phenotypic assessment.

## Methods

### Participants

The present sample included 77 patients who participated in a largely descriptive study of dyslipidemia and vascular atherosclerotic risk factors [2] plus a further 28 individuals recruited subsequently [5]. Collection of demographic and clinical data, as well as fasting metabolic blood measurements, were part of opportunistic clinical phenotyping. All investigations were undertaken under the auspices of ethically approved protocols with prior written informed consent of all participants.

### Assay methods

Thyroid hormones (free thyroxine [FT4], free triiodothyronine [FT3] and thyroid stimulating hormone [TSH]) were measured using a DELFIA® fluoroimmunometric assay (Wallac, Milton Keynes, UK). Reverse T3 (rT3) was measured by radioimmunoassay (Quest Laboratories, Heston, UK). Lipid profiles were measured by Advia Centaur (Siemens, Germany), NEFA by Roche Free Fatty Acids (Roche Diagnostics, Mannheim, Germany), and serum insulin by Diasorin XL Liason methods, with calculation of Homoeostasis Model Assessment (HOMA) IR from fasting plasma glucose and insulin concentrations using the following formula: HOMA-IR = fasting plasma glucose (mmol/L) × fasting plasma insulin (mU/L)/22.5, as described previously [6].

### Statistical analysis

The computer packages IBM SPSS Statistics 25 (IBM Corporation, Armonk, NY, USA) and StataSE 15 (College Station, TX: StataCorp LP) were used for statistical analysis. In this cross-sectional study, separate multivariable linear regression analyses were performed for the individual components of dyslipidemia (total serum cholesterol, LDL-cholesterol, HDL-cholesterol, triglycerides and total/HDL cholesterol ratio) as dependent variables. Age, sex, body mass index (BMI), fasting plasma glucose, fasting plasma NEFA and HOMA-IR, as well as TSH, FT4, FT3 and rT3 (which were all natural-log transformed because they exhibited right-skewed distributions) were included as candidate independent variables, with backward stepwise selection to determine the most parsimonious model. A two-tailed significance level of *P*<0.05 was used throughout.

## Results

### Participant characteristics

The present cohort comprised all 105 referred individuals with RTHβ (mean age 40.0 (range 15.7-67.8) years, 40% males) in whom fasting serum lipid concentrations available for analysis (see Table 1). One in nine were current smokers and most were in the overweight/obese body mass index (BMI) range. A small proportion were treated with a statin, consistent with a low prevalence (<2.0%) of known cardiovascular disease. One participant had co-incident type 1 diabetes. A majority (75.5%) had a raised HOMA-IR (>1) consistent with at least mild insulin insensitivity [6]. Just under a quarter had a clinically significant IR with a HOMA-IR of ≥2.5, a threshold based on a variety of studies of healthy adults of similar age to our participants [7–9]. The thyroid hormone profile of participants was consistent with RTHβ, with all circulating free T4 and free T3 concentrations above ranges in healthy controls (Table 1) in association with non-suppressed serum TSH concentrations. A small percentage (13.3%) of RTHβ patients were taking either carbimazole or thyroxine as part of their clinical management.

### Associates of lipid parameters

The results of multivariable analyses are summarized in Table 2. There were relatively few statistically significant independent variables associated with each of the lipid parameters. Age was positively related to serum total and LDL-cholesterol concentrations, while there were similar directional associations between BMI and serum total:HDL-cholesterol ratio, and HOMA-IR and serum triglycerides. The only thyroid function measure included as a significant independent variable in the individual models was reverse T3 which exhibited an inverse association with serum triglycerides. In all five regression models, less than a quarter of the variability in each measure was explained by the contributory independent variables.

## Discussion

The relationship between increasing age and serum LDL-cholesterol in the present young and middle-age adult participants with RTHβ is consistent with that found in general population studies [10]. Although serum LDL-cholesterol concentrations were significantly higher in the 77 individuals with RTHβ compared with age-, sex- and BMI-matched controls, as we reported previously [2], the difference between the mean values in each group was only 0.3 mmol/L. In addition, the absolute serum LDL-cholesterol concentration ranges in RTHβ participants and controls were similar (1.2-5.4 mmol/L versus 1.5-5.0 mmol/L) [2]. Our current observations suggest that a modest effect of hepatic end organ resistance on serum LDL-cholesterol in RTHβ, perhaps reflected by changes in hepatocyte expression of LDL receptors and thus circulating LDL-cholesterol clearance, is not paralleled by the magnitude of elevated thyroid hormone levels. This may indicate a degree of resistance within the hypothalamic-pituitary-thyroid axis. The significant positive associations between BMI and total:HDL-cholesterol ratio, and between HOMA-IR and serum triglycerides, in the present participants have also been observed in general population studies [4, 11]. These findings suggest that the recognized relationships between features of the Metabolic Syndrome [12] also prevail in RTHβ and are not significantly affected by the altered thyroid function status in this disorder.

The strong significant inverse relationship between reverse T3 and serum triglycerides is difficult to interpret. It has been suggested that, rather than being an inactive metabolite, reverse T3 might have biological activities, perhaps acting as a competitive inhibitor that disrupts T3 signalling by inhibiting deiodinase enzyme activity [13]. Since serum triglyceride concentrations can also be raised in individuals with conventional hyperthyroxinemia [14, 15], higher inhibitory reverse T3 concentrations may attenuate elevation of circulating triglycerides in RTHβ. Alternatively, it is possible that both the continuing production of ApoB contributing to raised serum triglycerides we have documented previously [2] and altered production of reverse T3 due to reduced hepatic activity of type 1 deiodinase (D1) (a recognised thyroid hormone regulated target gene) represent manifestations of hepatic refractoriness to thyroid hormone which change in parallel.

There was no significant association between plasma NEFA and serum triglycerides in the present participants, suggesting that, contrary to what has been hypothesisized previously [2], increased fatty acid availability does not contribute to RTHβ-associated hypertriglyceridemia. However, it is conceivable that the recognised day-to-day coefficient of variation in plasma NEFA levels of up to 50% could have masked an association with the single measurement of NEFA available in the present study [16].

3,5,3’-triiodothyroacetic acid (Triac), a thyroid hormone analogue with hepatic but not pituitary superagonist effects and a candidate therapy for RTHβ [17], has the potential to abrogate the modest increase in serum LDL-cholesterol (0.3 mmol/L [2]) seen in RTHβ and could also attenuate higher serum triglyceride concentrations. This would be at the expense of an even lower serum HDL cholesterol through direct modulation of hepatic lipoprotein metabolism independent of reduced circulating free thyroid hormone concentrations [18]. The present data showing no association between serum free T4 or T3 and lipid parameters in RTHβ suggest that Triac therapy would also exert similar effects in this disorder.

Increased rates of premature cardiovascular death associated with RTHβ [19] and the therapeutic potential of drugs such as Triac [17] call for a greater understanding of the mechanisms underlying RTHβ-associated dyslipidemia as a modifiable risk factor. We recently showed that, although absolute cardiovascular risk is modestly increased in RTHβ, non-ischemic myocardial dysfunction likely underlies early increased mortality with atherosclerotic disease contributing later in life [5]. Nevertheless, the management of insulin resistance, primarily through optimized lifestyle factors, would seem appropriate for patients with RTHβ [20] with consideration of lipid-modifying therapy based on conventional individual patient risk assessment and local guidelines [21, 22].

The present study had limitations. Since participants were referred to a specialist centre and RTHβ is known to be associated with a wide spectrum of clinical symptoms and phenotypes [23], it is conceivable that the present cohort is not wholly representative of the disorder in the wider population. We did not routinely measure waist and hip circumference which may have allowed a more precise and prognostically important anthropometric definition of visceral fat distribution than BMI [24, 25]. Although we had cardiovascular risk scores in the present participants [26], quantification of atheroma burden such as through coronary computed tomography angiography [27] could prove a more accurate prognostic tool in people with RTHβ-associated dyslipidemia.

In conclusion, the dyslipidemia associated with RTHβ shares recognized contributory factors revealed in general population studies but appears independent of thyroid hormone status. The reason for the significant inverse association between circulating reverse T3 and serum triglyceride concentrations is unclear and merits further investigation.

## Figures and Tables

**Table 1:** Characteristics of the participants with RTHβ.

Variable	n		Reference range
Age (years)	105	40.0±15.2	
Sex (% male)	105	40.0	
Smoking status (%):	104		
Never		64.4	
Ex-		24.0	
Current		11.5	
Weight (kg)	103	72.4±14.9	
Height (m)	103	1.65±0.10	
BMI (kg/m^2^)	105	26.4±4.9	
Fasting glucose (mmol/L)	99	5.0 [4.6-5.2]	
HbA_1c_ (mmol/mol)	61	35 [32-38]	
Serum insulin (pmol/L)	99	50 (26-97)	
HOMA-IR	94	1.6 (0.8-3.3)	
Total serum cholesterol (mmol/L)	102	4.9±1.1	
Serum HDL-cholesterol (mmol/L)	101	1.18±0.39	
Total:HDL cholesterol ratio	101	4.4±1.3	
Serum LDL-cholesterol (mmol/L)	101	3.0±0.9	
Serum triglycerides (mmol/L)	101	1.4 (0.8-2.3)	
Statin treatment (%)	104	6.7	
Plasma NEFA (μmol/L)	99	401 (259-621)	
Serum uric acid (mmol/L)	79	0.33±0.07	
History of myocardial infarction/stroke (%)	105	1.9	
eGFR <60 mL/min/1.73m^2^ (%)	78	0	
Serum TSH (mU/L)	105	2.6 (1.0-6.8)	0.7-4.5*
Serum free T4 (pmol/L)	97	31.8 (23.4-43.4)	9.7-17.3*
Serum free T3 (pmol/L)	103	11.3 (8.3-15.3)	3.4-7.0*
Serum reverse T3 (nmol/L)	97	0.62 (0.37-1.05)	0.12-0.36^†^
Carbimazole or thyroxine treatment (%)	105	13.3	

* mean ± 2SD for age, sex and BMI matched healthy controls from Moran *et al*. [2];

† in house reference range for healthy adults.

**Table 2:** Most parsimonious models of the components of dyslipidemia in adults with RTHβ. Only the statistically significant variables are shown for each component.

	Total serum cholesterol		Serum LDL-cholesterol		Serum HDL- cholesterol		Serum triglycerides		Total cholesterol:HDL- cholesterol ratio	
	B	*P*-value	B	*P*-value	B	*P*-value	B	*P*-value	B	*P*-value
Age (increase of 1 year)	0.035	<.001	0.027	<0.001						
BMI (increase of 1 kg/m^2^)									0.082	0.001
Ln(reverse T3) (pmol/L)*							-0.395	0.009		
Ln(HOMA-IR)*							0.310	0.007		
										
n	102		101		102		86		101	
Adjusted R^2^	0.224		0.201		0		0.126		0.091	

B = unstandardized regression coefficient;

* an increase of 1 in ln(x) equates to an increase of 2.718 in x

## Abbreviations

BMI: (body mass index)
FT3: (free triiodothyronine)
FT4: (free thyroxine)
HDL: (high-density lipoprotein)
HOMA: (Homoeostasis Model Assessment)
IR: (insulin resistance)
LDL: (low-density lipoprotein)
NEFA: (non-esterified fatty acid)
TSH: (thyroid stimulating hormone)
rT3: (reverse triiodothyronine)
RTHβ: (Resistance to Thyroid Hormone β)

## References

[1] Moran C, Schoenmakers N, Visser WE, Schoenmakers E, Agostini M, Chatterjee K (2022). Genetic disorders of thyroid development, hormone biosynthesis and signalling. Clin Endocrinol (Oxf).

[2] Moran C, McEniery CM, Schoenmakers N, Mitchell C, Sleigh A, Watson L (2021). Dyslipidemia, Insulin Resistance, Ectopic Lipid Accumulation, and Vascular Function in Resistance to Thyroid Hormone beta. J Clin Endocrinol Metab.

[3] Owen PJ, Chatterjee VK, John R, Halsall D, Lazarus JH (2009). Augmentation index in resistance to thyroid hormone (RTH). Clin Endocrinol (Oxf).

[4] Ormazabal V, Nair S, Elfeky O, Aguayo C, Salomon C, Zuniga FA (2018). Association between insulin resistance and the development of cardiovascular disease. Cardiovasc Diabetol.

[5] Davis TME, Davis WA, Moran C, Lyons G, Bryden E, Chatterjee K (2025). Cardiovascular Risk and Plasma N-terminal Pro-B-type Natriuretic Peptide in Adults With Resistance to Thyroid Hormone beta. J Endocr Soc.

[6] Petersen KF, Dufour S, Feng J, Befroy D, Dziura J, Dalla Man C (2006). Increased prevalence of insulin resistance and nonalcoholic fatty liver disease in Asian-Indian men. Proc Natl Acad Sci U S A.

[7] Ascaso JF, Pardo S, Real JT, Lorente RI, Priego A, Carmena R (2003). Diagnosing insulin resistance by simple quantitative methods in subjects with normal glucose metabolism. Diabetes Care.

[8] Tahapary DL, Pratisthita LB, Fitri NA, Marcella C, Wafa S, Kurniawan F (2022). Challenges in the diagnosis of insulin resistance: Focusing on the role of HOMA-IR and Tryglyceride/glucose index. Diabetes Metab Syndr.

[9] Biernacka-Bartnik A, Kocelak P, Owczarek AJ, Choreza PS, Markuszewski L, Madej P (2023). The cut-off value for HOMA-IR discriminating the insulin resistance based on the SHBG level in women with polycystic ovary syndrome. Front Med (Lausanne).

[10] Downer B, Estus S, Katsumata Y, Fardo DW (2014). Longitudinal trajectories of cholesterol from midlife through late life according to apolipoprotein E allele status. Int J Environ Res Public Health.

[11] Brenner DR, Tepylo K, Eny KM, Cahill LE, El-Sohemy A (2010). Comparison of body mass index and waist circumference as predictors of cardiometabolic health in a population of young Canadian adults. Diabetol Metab Syndr.

[12] Cornier MA, Dabelea D, Hernandez TL, Lindstrom RC, Steig AJ, Stob NR (2008). The metabolic syndrome. Endocr Rev.

[13] Friberg L, Drvota V, Bjelak AH, Eggertsen G, Ahnve S (2001). Association between increased levels of reverse triiodothyronine and mortality after acute myocardial infarction. Am J Med.

[14] Nikkila EA, Kekki M (1972). Plasma triglyceride metabolism in thyroid disease. J Clin Invest.

[15] Cachefo A, Boucher P, Vidon C, Dusserre E, Diraison F, Beylot M (2001). Hepatic lipogenesis and cholesterol synthesis in hyperthyroid patients. J Clin Endocrinol Metab.

[16] Frayn KN (2005). Plasma non-esterified fatty acids: why are we not measuring them routinely?. Ann Clin Biochem.

[17] Groeneweg S, Peeters RP, Visser TJ, Visser WE (2017). Therapeutic applications of thyroid hormone analogues in resistance to thyroid hormone (RTH) syndromes. Mol Cell Endocrinol.

[18] Sherman SI, Ladenson PW (1992). Organ-specific effects of tiratricol: a thyroid hormone analog with hepatic, not pituitary, superagonist effects. J Clin Endocrinol Metab.

[19] Okosieme OE, Usman D, Taylor PN, Dayan CM, Lyons G, Moran C (2023). Cardiovascular morbidity and mortality in patients in Wales, UK with resistance to thyroid hormone beta (RTHbeta): a linked-record cohort study. Lancet Diabetes Endocrinol.

[20] Cohn G, Valdes G, Capuzzi DM (2001). Pathophysiology and treatment of the dyslipidemia of insulin resistance. Curr Cardiol Rep.

[21] Smith DA (2007). Treatment of the dyslipidemia of insulin resistance. Med Clin North Am.

[22] Grundy SM, Stone NJ, Bailey AL, Beam C, Birtcher KK, Blumenthal RS (2019). 2018 AHA/ACC/AACVPR/AAPA/ABC/ACPM/ADA/AGS/APhA/ASPC/NLA/PCNA Guideline on the Management of Blood Cholesterol: A Report of the American College of Cardiology/American Heart Association Task Force on Clinical Practice Guidelines. Circulation.

[23] Olateju TO, Vanderpump MP (2006). Thyroid hormone resistance. Ann Clin Biochem.

[24] Khan I, Chong M, Le A, Mohammadi-Shemirani P, Morton R, Brinza C (2023). Surrogate Adiposity Markers and Mortality. JAMA Netw Open.

[25] Krakauer NY, Krakauer JC (2018). Anthropometrics, Metabolic Syndrome, and Mortality Hazard. J Obes.

[26] Hippisley-Cox J, Coupland C, Brindle P (2017). Development and validation of QRISK3 risk prediction algorithms to estimate future risk of cardiovascular disease: prospective cohort study. BMJ.

[27] Nurmohamed NS, Min JK, Anthopolos R, Reynolds HR, Earls JP, Crabtree T (2024). Atherosclerosis quantification and cardiovascular risk: the ISCHEMIA trial. Eur Heart J.

